# The morphology of the inner ear of squamate reptiles and its bearing on the origin of snakes

**DOI:** 10.1098/rsos.170685

**Published:** 2017-08-23

**Authors:** Alessandro Palci, Mark N. Hutchinson, Michael W. Caldwell, Michael S. Y. Lee

**Affiliations:** 1 South Australian Museum, Adelaide, South Australia, Australia; 2 College of Science and Engineering, Flinders University, Adelaide, South Australia, Australia; 3 School of Biological Sciences, University of Adelaide, Adelaide, South Australia, Australia; 4 Department of Biological Sciences, University of Alberta, Edmonton, Alberta, Canada

**Keywords:** ecology, evolution, labyrinth, geometric morphometrics, principal components analysis, canonical variates analysis

## Abstract

The inner ear morphology of 80 snake and lizard species, representative of a range of ecologies, is here analysed and compared to that of the fossil stem snake *Dinilysia patagonica*, using three-dimensional geometric morphometrics. Inner ear morphology is linked to phylogeny (we find here a strong phylogenetic signal in the data that can complicate ecological correlations), but also correlated with ecology, with *Dinilysia* resembling certain semi-fossorial forms (*Xenopeltis* and *Cylindrophis*), consistent with previous reports. We here also find striking resemblances between *Dinilysia* and some semi-aquatic snakes, such as *Myron* (Caenophidia, Homalopsidae). Therefore, the inner ear morphology of *Dinilysia* is consistent with semi-aquatic as well as semi-fossorial habits: the most similar forms are either semi-fossorial burrowers with a strong affinity to water (*Xenopeltis* and *Cylindrophis*) or amphibious, intertidal forms which shelter in burrows (*Myron).* Notably, *Dinilysia* does not cluster as closely with snakes with exclusively terrestrial or obligate burrowing habits (e.g. scolecophidians and uropeltids). Moreover, despite the above similarities, *Dinilysia* also occupies a totally unique morphospace, raising issues with linking it with any particular ecological category.

## 1. Introduction

The debate on the ecological driver(s) that led to the origin of snakes is one of the most long-lasting and controversial in biology and evolution. There are currently two main scenarios that view snakes either as deriving from worm-like burrowers (e.g. [1,2]) or from eel-like swimmers (e.g. [3,4]), and these hypotheses are often labelled as the ‘burrowing origin scenario’ and the ‘aquatic origin scenario’. One of the most recent and novel contributions to this debate [2] favoured burrowing origins, based on similarities between the inner ear of the basal fossil snake *Dinilysia patagonica* and that of a broad range of burrowing living squamates (i.e. lizards, amphisbaenians and snakes).

To test the hypothesis and conclusions of [2], we generated an expanded dataset consisting of 81 digital endocasts of the inner ear of squamate reptiles (about twice as many species as those in the earlier study), and refined the landmarking scheme and ecological categories. The data were subjected to a variety of statistical analyses that produced very different results from those of [2]. In this study, the implications of our finding on the origin and early evolution of snakes, and in particular the possible ecologies of *Dinilysia*, are discussed at length. We also examine the epistemic issues related to drawing general inferences on the evolution of this group of reptiles based on a single extinct species.

## 2. Material and methods

High-resolution computer tomographies (micro-CT) of the heads of 72 squamate reptiles were acquired using a Skyscan 1076 at Adelaide Microscopy (University of Adelaide, Adelaide, South Australia) (electronic supplementary material S1, table S1; see this table also for list of taxonomic authorities) and the software NRecon (Bruker microCT) was used to reconstruct stacks of images (.bmp) from the micro-CT scan data. Digital data (micro-CT scan images) of seven specimens (*Anomochilus leonardi*, *Bipes biporus*, *Calabaria reinhardtii*, *Eryx colubrinus*, *Loxocemus bicolor*, *Python molurus*, and *Tropidophis haetianus*) were obtained courtesy of Drs Olivier Rieppel and Jessie Maisano (Deep Scaly Project; electronic supplementary material S1, table S1). These data were then visualized in the software Avizo v. 9.0 (Konrad-Zuse-Zentrum für Informationstechnik Berlin and Visualization Sciences Group), where a digital endocast of the right inner ear was produced for each specimen via segmentation. These digital endocasts were then exported as ‘.ply’ files, a format that is suitable for landmarking in the program Landmark Editor v. 3.6 [5].

Digital endocasts (‘.ply’ format) of the fossil squamates *Dinilysia* and *Platecarpus* were available from the supplementary data in [2] (MorphoBank online repository, project 2170 [6]). We did not use other endocasts from that particular study because most digital models had low resolution and the lagenar portion was often missing (not segmented), so we opted for sampling our own set of taxa instead. Moreover, by sampling a new set of specimens and taxa we could provide a stronger test of the ecological affinity of the fossil snake *Dinilysia*.

**Figure 1.**
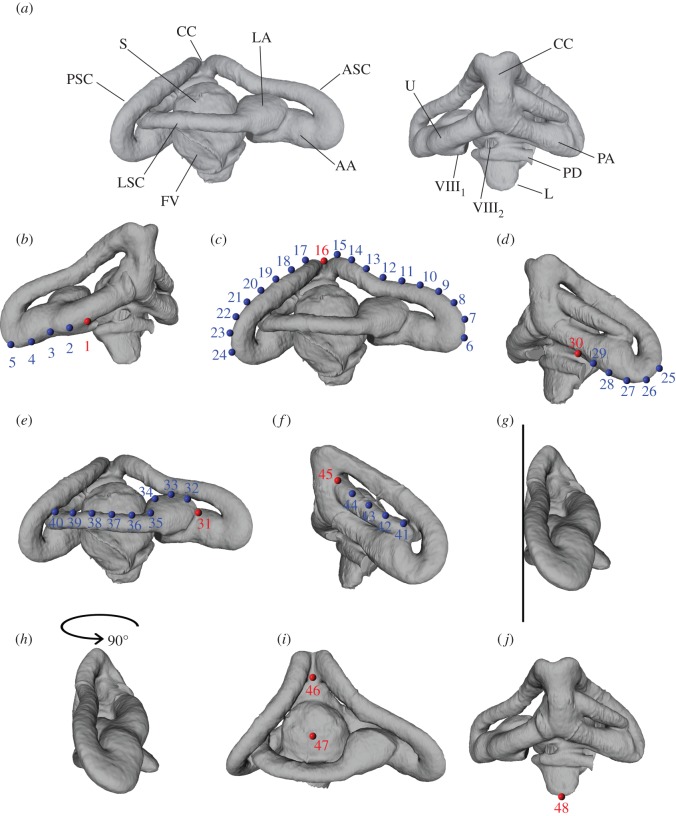
Digital endocast of the right inner ear of *Lycodon aulicus* (SAMA R36823). (*a*) Main anatomical regions of the inner ear of a squamate reptile (the colubroid snake *Lycodon*), lateral view to the left, and medial view to the right; (*b–j*) steps followed while landmarking the specimens; see text for details. Abbreviations: AA, anterior ampulla; ASC, anterior semicircular canal; CC, common crus; FV, fenestra vestibuli (=fenestra ovalis); L, lagena (= cochlea); LA, lateral ampulla; LSC, lateral (= horizontal) semicircular canal; PA, posterior ampulla; PD, perilymphatic duct (part); PSC, posterior semicircular canal; S, sacculus; U, utriculus; VIII_1_, anterior branch of the auditory nerve (part); VIII_2_, posterior branch of the auditory nerve (part).

All digital endocasts (81 species) were landmarked in Landmark Editor v. 3.6 [5], following the pattern shown in figure 1 and the procedure reported below.

Anatomical terms follow [7,8]. The first landmark (landmark 1) was fixed and placed at the notch marked by the point where a branch of the auditory nerve (anterior branch of the VIII nerve) [7] meets the utriculus (figure 1*b*). This was followed by a series of sliding semilandmarks, spaced as evenly as possible, along the anterior semicircular canal, on the line of maximum curvature facing away from the sacculus (figure 1*a–c*). The landmark located on the top of the crus communis was a fixed landmark (landmark 16). A second series of evenly spaced sliding semilandmarks was placed along the posterior semicircular canal, again along the line of maximum curvature facing away from the sacculus (figure 1*c*,*d*). This series terminated in a fixed landmark (landmark 30) located at a point where the posterior ampulla meets the sacculus, typically dorsal to the start of the perilymphatic duct (the canal that leads to the fenestra rotunda), and at a point where the ampulla is considerably tapered posteriorly. The next landmark (landmark 31) was also fixed and located on the dorsal end of the groove that separates lateral and anterior ampulla, with the lateral semicircular canal placed horizontally (figure 1*e*). This was followed by a series of evenly spaced sliding semilandmarks along the lateral semicircular canal in a posterior direction and located dorsolaterally along the canal. This series terminated in a fixed landmark (landmark 45) located at the posterior end of the lateral semicircular canal, which is typically marked by a more or less pronounced concavity at the base of the crus communis (figure 1*f*). The next two landmarks were fixed, and placed after the digital endocast of the inner ear was positioned following two simple steps: (i) the semicircular canals were aligned tangent to an imaginary vertical plane located to the left of the endocast (figure 1*g*) and (ii) the endocast was rotated about 90° counterclockwise (as seen from a dorsal perspective), so that the lateral surface of the sacculus was facing the observer (figure 1*h*,*i*). This step ensured that the sacculus was observed from a consistent angle when placing the next two landmarks. Landmark 46 was placed at the ventral end of the groove that separates anterior and posterior semicircular canals at the level of the crus communis; while landmark 47 was placed approximately in the centre of the sacculus, which seen in projection from such angle can usually be approximated to a circle. When this was not the case (i.e. the sacculus was far from hemispherical) the landmark was placed in the circumcentre, i.e. in a point that would correspond to the centre of the circumscribed circle that best fits the outline of the sacculus in the specific view obtained following steps 1 and 2. The last fixed landmark (landmark 48) was placed at the bottom of the lagena, typically at the point of maximum curvature, but excluding sutures, since the latter can create an artificial relief (figure 1*j*).

It should be noted that in some species the posterior semicircular canal partially intersects the lateral semicircular canal. This can create problems in the placing of evenly spaced semilandmarks along the lateral semicircular canal (landmarks 32–44). We avoided placing a semilandmark medial to the intersection (i.e. medial to the posterior semicircular canal), and placed one semilandmark anterior to the intersection and another posterior to it. This was considered to be the best solution, because with the adoption of sliding semilandmarks the spacing does not have to be strictly even, as long as the general curvature defined by these points remains representative of the general curvature of the shape that they are meant to represent [9].

The semilandmarks were approximately evenly spaced between pairs of fixed landmarks, but the actual spacing varied between species (e.g. some species have a much longer anterior semicircular canal relative to others, which nonetheless needs to accommodate the same number of semilandmarks).

The landmark configurations of all specimens are provided in electronic supplementary material S2 (‘.nts’ format). The landmark configurations (eight fixed landmarks, 40 sliding semilandmarks) were then scaled and aligned with a Procrustes superimposition using the R v. 3.3.2 [10] package geomorph v. 3.0.3 [11]. Analyses of the dataset were carried out in R v. 3.3.2 [10] using the packages geomorph v. 3.0.3 [11], Morpho v. 2.4.1.1 [12], phytools [13] and phylotools v. 0.1.2 [14], and in MorphoJ v. 1.06d [15]. Our analysis included an evaluation of the magnitude of the error due to stochastic inconsistencies in the placement of the landmarks; this was done by placing landmarks on five randomly selected species five times and then plotting the Procrustes-aligned configurations in shape space using principal components analysis (PCA)

We assessed the effect of phylogenetic signal using the function ‘physignal’ in the package geomorph v. 3.0.3 [11], using a phylogeny with branch lengths and divergence times modified (i.e. pruned of unsampled terminal taxa) from [16], and two phylogenies where the two fossil taxa *Platecarpus tympaniticus* and *Dinilysia patagonica* had been inserted into the previous phylogeny based on [3,17] and [18–20], respectively. The two trees with fossil taxa were used to take into account the uncertainty regarding the placement of *Dinilysia* [3,17–20] (see below for details). Phylogenetic signal was tested using both the phylogeny of extant taxa and the two trees inclusive of all taxa (79 and 81 taxa, respectively). The test was performed with 1000 random permutations

We carried out non-phylogenetic and phylogenetic Procrustes analyses of variance (ANOVA) using a randomized residual permutation procedure (1000 iterations) [21–23] to test for correlation between shape and groups defined based on ecological preference. The phylogenetic ANOVA was run only using the tree of 79 extant species, because that is the tree with less uncertainty about relationships and ecological data are available for all taxa.

We used an ordinary (i.e. non-phylogenetic) PCA to see where *Dinilysia* is located in shape space compared to other taxa based solely on morphology. We also carried out a phylogenetically informed principal components analysis (phylogenetic PCA, or PPCA) to provide a correction for the distribution in shape space of the taxa that may be affected by phylogenetic signal. The phylogenetic PCA was performed using the R package phytools v. 0.6-00 (function phyl.pca) [13,24]. This function relies on a model of evolution and the two options are uniform Brownian motion (BM) or Pagel's lambda (lambda). The model selected was BM, because the function was unable to analyse our dataset under the lambda model (too many variables).

We tested for correlation between centroid size (CS, an index of overall size) [25] and first principal component (PC1; from both ordinary and phylogenetic PCA) using Pearson, Kendall and Spearman methods [26]. We included a classification (group affinity) test using the ‘typprobClass’ function in the package Morpho v. 2.4.1.1 [12] to find which ecological group *Dinilysia* is closest to based on its first two principal components (PCs) scores (tests on scores from both ordinary and phylogenetic PCA; groups were defined for all taxa except *Dinilysia*).

A canonical variates analysis (CVA) was used to display how strongly defined the groups are in shape space. This analysis was first run in R using the package Morpho v. 2.4.1.1 [12] with jacknife cross-validation (1000 replicates), and then plots and diagrams were produced in MorphoJ [14]. We are not aware of the possibility of running a phylogenetically informed CVA analysis of three-dimensional landmark coordinates in either MorphoJ v.1.06d [14] or the R packages geomorph v. 3.0.3 [11] and Morpho v. 2.4.1.1 [12]. Nevertheless, we think that the results of an ordinary CVA can be useful in describing general patterns of shape change between taxa in different ecological categories, keeping in mind the caveat that in this plot shape is also in part affected by phylogenetic constraints.

The phylogenetic tree adopted for the various phylogenetic tests (phylogenetic signal, phylogenetic ANOVA, and phylogenetic PCA) using extant taxa was obtained from [16] after pruning unsampled species in Mesquite v. 3.2 [27] and retaining branch lengths. Two additional trees inclusive of the fossil taxa *Platecarpus tympaniticus* and *Dinilysia patagonica* were obtained after insertion of these fossils into the previous tree using the editing tools of Mesquite v. 3.2 [27]. The fossils were first positioned according to the phylogeny in [17], and in particular the branch of *Platecarpus tympaniticus* was inserted halfway between the node representing the most recent common ancestor (MRCA) of extant snakes (Ophidia) and that of the clade ((Anguimorpha, Iguania), Ophidia); while the branch of *Dinilysia patagonica* was inserted halfway between the nodes representing the MRCA of *Liotyphlops* and *Anilius* and the MRCA of Alethinophidia (i.e. MRCA of *Anilius* and *Causus*) (electronic supplementary material S3, figure S1*a*). Considering the phylogenetic uncertainty regarding the placement of our main taxon of interest (*Dinilysia*) [3,17–20], we also ran a series of phylogenetically informed analyses based on a tree where this taxon was basal to all extant snakes (Ophidia) (electronic supplementary material S3, figure S1*b*). The tip ages of the fossils were based on [28,29] for *Platecarpus tympaniticus* and *Dinilysia patagonica*, respectively. It should be noted that the specimen of *Platecarpus* in [2,6] is referred to as *P.* ‘*coryphaeus*’, but that is a junior synonym of *P. tympaniticus* [30,31]. It should also be noted that even if vertebrae of *Dinilysia* sp. have been reported from the Campanian of Argentina [32], the inner ear endocast from [2,6] belongs to *Dinilysia patagonica* proper, and that specimen cannot be younger than the upper Santonian [29]. Therefore, *Platecarpus tympaniticus* was assigned a tip age of 81 million years [28], while *Dinilysia patagonica* was assigned a tip age of 83.6 million years [29,33].

Estimates of the ecological preferences of all species (except *Dinilysia*, which we left as unknown) were obtained from a survey of the literature (electronic supplementary material S4, table S2). To improve comparisons we expanded the three categories of [2] into five categories: (i) generalist, for those squamates that do not show any particular preference for a particular habitat and commonly forage on the surface of the ground; (ii) arboreal, for those species that spend most of their time basking and foraging in trees or shrubs; (iii) fossorial, for those species that tend to spend a considerable amount of their time underground in burrows or that forage under loose soil and vegetation; (iv) aquatic, for those species that spend most or all of their time in an aquatic environment and often show anatomical specializations for swimming (e.g. sea snakes, mosasaurs); and (v) semi-aquatic, for those squamates that spend considerable amounts of time in the water, but often emerge to feed, bask, or reproduce (e.g. *Eunectes*, most homalopsids, *Natrix*, *Intellagama*).

These categories are all rather loose and overlap—placement of many species could have been in more than one of the categories—so should not be regarded as unambiguous. This is especially true for the ‘fossorial’ category (henceforth fossorial *sensu lato*, s.l.), because the terms ‘fossorial’ and ‘burrower’ are often used very loosely by authors to refer to active obligate burrowers (e.g. amphisbaenians), semi-fossorial/cryptic taxa (e.g. *Loxocemus*), as well as surface dwelling forms that can dig for prey (e.g. *Aspidites*). These approximations also, unfortunately, critically impair our ability to characterize the ‘burrowing origins scenario’ both empirically and hypothetically [3].

The primary ecological points of interest are ultimately those of the species that have an inner ear morphology similar to that of *Dinilysia*. For these species, we have presented the particular ecological information that is available in the literature. Additional notes on assignment of species to categories are provided in electronic supplementary material S4, table S2. The R scripts and settings used for our analyses are available in electronic supplementary material S5.

## 3. Results

The PCA of the five randomly selected species, each landmarked five times in order to estimate the magnitude of the error introduced by inconsistencies in landmark placement, shows that the selected landmarking scheme is robust and produces only small stochastic variations in the placement of the taxa in shape space. The spread of the points was always smaller than 0.030 along PC1 and smaller than 0.025 along PC2 (electronic supplementary material S3, figure S2).

The tests for phylogenetic signal rejected the null hypothesis regardless of the inclusion of fossils and the placement of *Dinilysia* (*H*_0_ = no phylogenetic signal present [34]) (*K* = 0.4008, *p* = 0.001 when fossils were excluded; *K* = 0.4047, *p* = 0.001 when fossils were included and *Dinilysia* is a stem alethinophidian; *K* = 0.4112, *p* = 0.001 when fossils were included and *Dinilysia* is a stem ophidian), which implies that the shape data are also affected by evolutionary history. A value of the *K* statistic much lower than 1 would suggest that the evolution of the inner ears of squamates did not closely conform to a uniform Brownian motion model of evolutionary change [34].

The non-phylogenetic Procrustes ANOVA found a statistically significant correlation between shapes and grouping based on ecological preferences (the null hypothesis—*H*_0_—of no difference between the group means was rejected; *F*_79_ = 3.43, *p*-value = 0.001). This implies that, when phylogenetic relationships are not factored in, the variability within the ecological groups is significantly less than that among different groups [19].

Because the test for phylogenetic signal rejected the null hypothesis, we also ran a phylogenetically informed Procrustes ANOVA [20]. This analysis was based on the tree containing only extant taxa, and found that, despite the presence of phylogenetic signal in the data, there is still a significant correlation between shape and ecological groups (the null hypothesis—*H*_0_—of no relationship between shape and groups was rejected; *F*_78_ = 4.37; *p*-value = 0.003). A limitation of this method is that it is based on a Brownian motion model of evolution (the only model currently available for Procrustes ANOVA using multivariate data [20]), and as mentioned above this model may not accurately describe the evolution of the inner ear of squamate reptiles. However, a Brownian motion model of evolution, although not ideal, still represents a reasonable approximation in the absence of a better more parameter-rich model [20].

**Figure 2.**
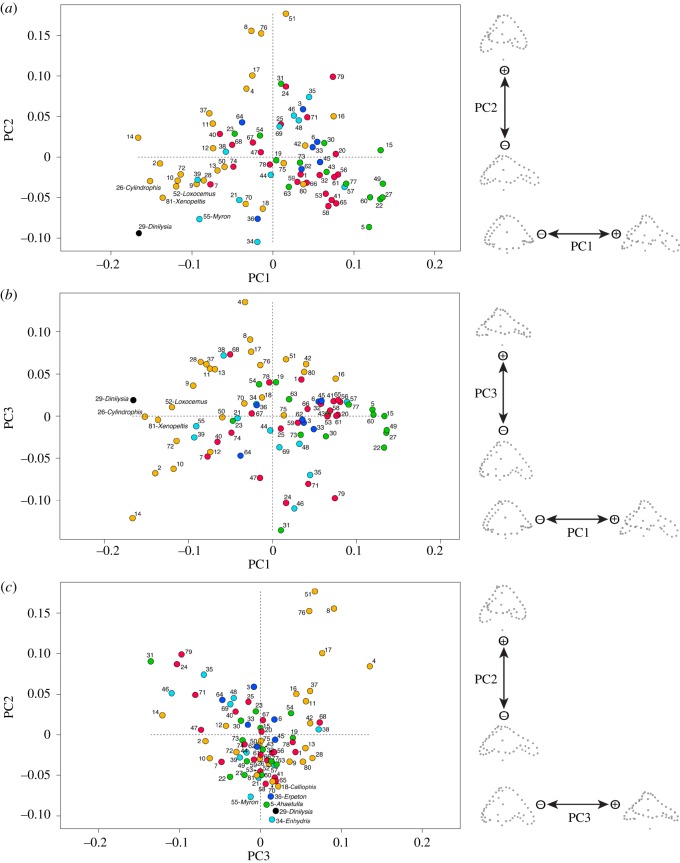
Distribution of the selected taxa in the morphospace of inner ears plotted against the first three principal components (ordinary PCA). Note how *Dinilysia* (black dot) occupies a position on the periphery of the main cluster in all plots. (*a*) PC2 plotted against PC1; (*b*) PC3 plotted against PC1; (*c*) PC2 plotted against PC3. Diagrams to the right are projections of the Procrustes landmark configurations towards the positive and negative extremes of each axis; all projections are in lateral view, anterior to the right. Orange colour indicates fossorial (s.l.) taxa; cyan indicates semi-aquatic taxa; blue indicates fully aquatic taxa; green indicates arboreal taxa; and red indicates generalist taxa. The names of the taxa that are closest to *Dinilysia* in each plot are shown; for all other taxa see electronic supplementary material S4, table S2, where species names are provided for all numbers.

The ordinary PCA inclusive of all species (figure 2) produced 81 PCs, the first three of which account for 51% of the total variance (electronic supplementary material S3, figure S3). The plots of the first three PCs are discussed below, but values for the first 10 PCs are available in electronic supplementary material S6, table S3. An interactive three-dimensional plot of PC1 versus PC2 versus PC3 with the same colour-coding as in figure 2 is available in electronic supplementary material S7.

Shape changes along the negative direction of PC1 correspond to an overall enlargement of the saccular region and semicircular canals that are more closely adpressed to the sacculus (especially noticeable in the anterior semicircular canal, which projects anteriorly further away along positive values of PC1). Changes along the negative direction of PC2 correspond to an overall antero-posterior stretching of the whole inner ear cavity, and a shortening of the lagena (figure 2). Changes in the negative direction of PC3 correspond to a dorsoventral expansion of the sacculus and semicircular canals; the expansion does not affect, however, the ventral extent of the lagena.

In the plot of PC1 versus PC2 the fossil snake *Dinilysia* falls in the extreme lower left, which corresponds to the most negative values of both PC1 and PC2, quite separate from all other taxa. The closest taxa are the snakes *Cylindrophis*, *Xenopeltis*, *Loxocemus*, and *Myron*; as noted, *Cylindrophis* and *Xenopeltis* are semi-fossorial snakes from South-East Asia with a predilection for wet environments (e.g. rivers, swamps, rice fields) [35], while *Myron* is a semi-aquatic snake that lives in mangrove flats, creeks and estuaries of northern Australia and New Guinea [36]. *Loxocem*us is a semi-fossorial snake that does not actively dig burrows, but hides in loose soil and leaf litter [35]. It is important to note that there is considerable overlap of squamates with different ecologies in this plot, but generalist forms tend to stay clustered around the centre, while fossorial and arboreal forms are the only ones that spread, respectively, to the far left (PC1 strongly negative) and far right (PC1 strongly positive) sides of the plot. The obligate burrowing snakes that are representative of the ‘Scolecophidia’ (all except *Acutotyphlops*) occupy a part of morphospace that is quite distinct from all other snakes, with very high values of PC2, and values of PC1 that are close to the mean (PC1 = 0.0).

In the plot of PC1 versus PC3 the fossil snake *Dinilysia* is placed closest to the semi-fossorial snakes *Cylindrophis* and *Xenopeltis* (as in PC1 versus PC2), but the semi-aquatic homalopsid snakes *Myron* and *Fordonia* are not far away (figure 2). No obligate burrowing forms occupy the bottom right shape space quadrant, where PC1 values are positive and PC3 values are negative.

In the plot of PC2 versus PC3 *Dinilysia* falls at the bottom of the cluster, surrounded by aquatic (*Erpeton*) and semi-aquatic (*Myron* and *Enhydris*) snakes as well as one arboreal taxon (*Ahaetulla*). In this plot most species tend to be closely clustered together, with the notable exception of some obligate burrowing forms (mostly scolecophidians), which are scattered in the top right quadrant corresponding to positive values of PC2 and PC3.

**Figure 3.**
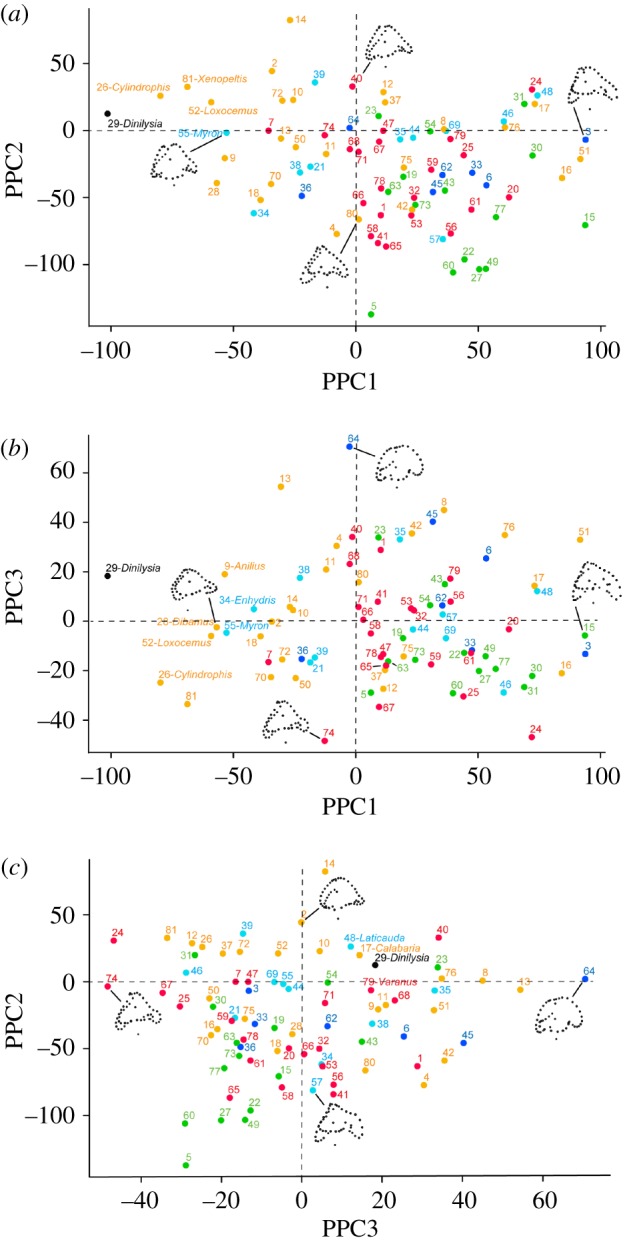
Distribution of the selected taxa in the morphospace of inner ears plotted against the first three phylogenetic principal components (PPCs). Note how *Dinilysia* (black dot) occupies a position on the periphery of the main cluster in all plots except (*c*). (*a*) PPC2 plotted against PPC1; (*b*) PPC3 plotted against PPC1; (*c*) PPC2 plotted against PPC3. Diagrams are projections of the Procrustes landmark configurations of actual specimens located towards the positive and negative extremes of each axis and as close as possible to the origin of the orthogonal axis; all projections are in lateral view, anterior to the right. Colour coding is the same as in figure 2. The names of the taxa that are closest to *Dinilysia* in each plot are shown; for all other taxa see electronic supplementary material S4, table S2, where species names are provided for all numbers.

The phylogenetic PCA is a rigid rotation of the ordinary PCA axis to better represent distances between specimens in accordance to a phylogenetic tree and an evolutionary model [37]. This rotation causes the scatterplots on the phylogenetic PCs (PPCs) to look quite different from those on the ordinary PCs. However, not much changes regarding the isolation of *Dinilysia* from all other taxa and the species that are morphologically the closest (figure 3), regardless of which tree is used. The plots of the PPCs based on the tree with *Dinilysia* as a stem ophidian are almost identical (results not shown, but first 10 PPCAs for both analyses are available in electronic supplementary material S8, table S4). Most of the variance is explained by the first three PPCs (approx. 57%; regardless of the tree used; electronic supplementary material S3, figure S4). In the plot of PPC1 versus PPC2, *Dinilysia* is still placed close to the semi-fossorial snakes *Cylindrophis*, *Loxocemus* and *Xenopeltis*, as well as the semi-aquatic *Myron*; in the plot of PPC1 versus PPC3, *Dinilysia* is very far from all taxa, and the closest taxa are the semi-fossorial squamates *Anilius*, *Cylindrophis*, *Dibamus* and *Loxocemus*, as well as the semi-aquatic snakes *Enhydris* and *Myron*; in the plot of PPC3 versus PPC2, the position of *Dinilysia* is quite different from that shown by ordinary PC3 versus PC2. Here this taxon is placed among species of various ecologies, and the closest are the semi-fossorial snake *Calabaria*, the semi-aquatic snake *Laticauda*, and the generalist lizard *Varanus*.

Unlike in the ordinary PCA, the points on the PPCA plots look much more evenly spread out in shape space. Similarly to the ordinary PCA, however, all ecological groups still tend to overlap. Interestingly, no arboreal forms have negative values of PPC1, probably due to their expanded anterior semicircular canal (figure 3).

In figure 3 landmark configurations representative of positive and negative extremes of each PPC axis are not shown because in these plots the transformed data are not the actual shapes, but shape differences from the estimated root standardized by branch lengths [37]. This makes mental interpolation between extreme configurations non-intuitive; therefore we decided to add to the plots actual configurations of specimens that fall approximately on opposite sides along each PPC and across from the origin (which, again, unlike ordinary PCA does not represent the average Procrustes landmark configuration, but the configuration of the hypothetical ancestor reconstructed at the most basal node of the tree [37]). A three-dimensional plot of the first three PPCAs is available in electronic supplementary material S9.

The tests for a possible correlation between both ordinary and phylogenetic PC1s and centroid size (an index of overall size) [22] could not reject the null hypothesis of no association between the tested variables (i.e. *H*_0_ = there is no correlation between the two tested variables that differs from what we might expect to occur randomly) [23] under all alternative methods (for correlation with ordinary PC1, Pearson's method: *t*_79_ = 0.696, cor = 0.078, *p* = 0.488; Kendall's method: *z* = 1.93, *τ* = 0.146, *p* = 0.053; Spearman's method: *S* = 70674, *ρ* = 0.202, *p* = 0.071—for correlation with phylogenetic PC1 and *Dinilysia* as a stem alethinophidian, Pearson's method: *t*_79_ = −0.3614, cor = −0.041, *p* = 0.719; Kendall's method: *z* = −1.21, *τ* = −0.092, *p* = 0.224; Spearman's method: *S* = 100770, *ρ* = −0.138, *p* = 0.219—for correlation with phylogenetic PC1 and *Dinilysia* as a stem ophidian, Pearson's method: *t*_79_ = −0.3557, cor = −0.040, *p* = 0.723; Kendall's method: *z* = −1.17, *τ* = −0.088, *p* = 0.243; Spearman's method: *S* = 100360, *ρ* = −0.133, *p* = 0.235). This means that overall size has no statistically significant effect on the values of both ordinary and phylogenetic PC1.

We then tested in which group *Dinilysia* would be classified based on its PCs scores from the ordinary and phylogenetic PCAs (first 2 PCs) using the typprobClass function in the package Morpho v. 2.4.1.1 [12]. This method produces the typicality probability that a given specimen belongs to each of the defined groups based on its distance from the group means. As a given specimen can fall within multiple groups (or none of them), these probabilities do not necessarily sum to 1. Using this method and the first two PCs *Dinilysia* was most plausibly classified as a fossorial (s.l.) snake, semi-aquatic was not far behind, and all probabilities were low regardless of phylogenetic correction and tree adopted. The results based on the ordinary PCA were *p* = 0.044 for fossorial, *p* = 0.020 for semi-aquatic, *p* = 0.005 for generalist, *p* = 0.004 for aquatic, and *p* = 0.001 for arboreal. There was a very high probability (0.98) that *Dinilysia* fits none of these groups—i.e. falls outside of the group inclusive of all defined groups (a second analysis was run where all snakes except *Dinilysia* were assigned to the same group). The results based on first two PCs of the phylogenetic PCA are not too dissimilar regardless of whether *Dinilysia* is considered a stem alethinophidian or a stem ophidian. When *Dinilysia* is a stem alethinophidian: *p* = 0.049 for fossorial, *p* = 0.018 for semi-aquatic, *p* = 0.007 for generalist, *p* = 0.003 for aquatic, and *p* = 0.001 for arboreal. When *Dinilysia* is a stem ophidian: *p* = 0.058 for fossorial, *p* = 0.021 for semi-aquatic, *p* = 0.008 for generalist, *p* = 0.003 for aquatic, and *p* = 0.001 for arboreal. The probability that *Dinilysia* does not belong to any group is again very high regardless of the position of this fossil (0.98 when *Dinilysia* is a stem alethinophidian, and 0.97 when it is a stem ophidian).

**Figure 4.**
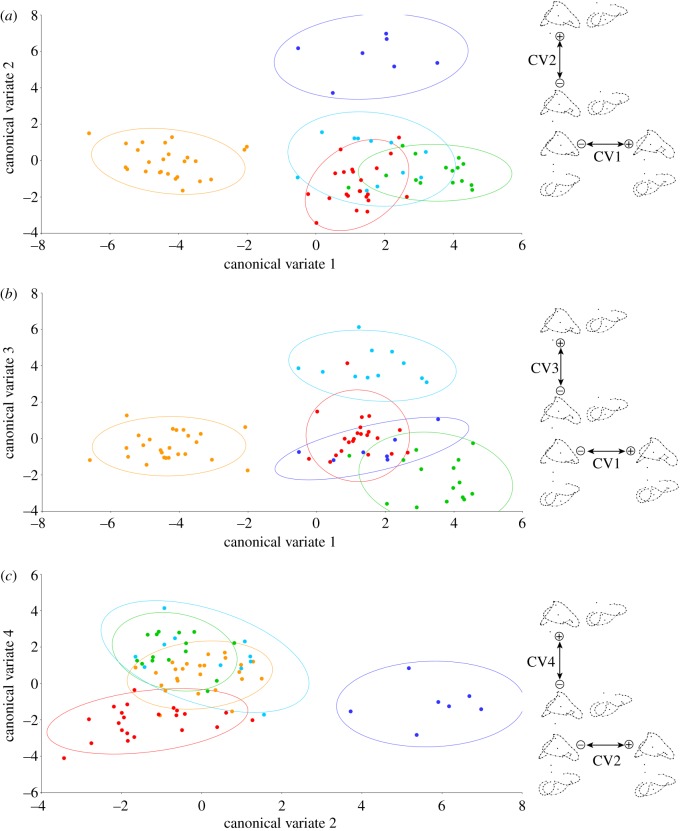
Distribution of 80 taxa (all except *Dinilysia*, whose ecology is unknown) in the morphospace of inner ears plotted against the first four canonical variates (ordinary CVA). (*a*) CV2 plotted against CV1; (*b*) CV3 plotted against CV1; (*c*) CV4 plotted against CV2. Orange colour indicates fossorial (s.l.) taxa; cyan indicates semi-aquatic taxa; blue indicates fully aquatic taxa; green indicates arboreal taxa; and red indicates generalist taxa. 90% confidence ellipses for each ecological group are also shown. Diagrams to the right are projections of the Procrustes landmark configurations towards the positive and negative extremes of each axis, in lateral and dorsal (to the right or below the former) views; anterior is to the right in all projections.

Ordinary CVA was used to illustrate the strength of the separation of the different ecological groups in shape space, and to detect which morphological changes define the different groups. With five groups, all variance in the dataset was described by four canonical variates (CV1 = 50.39%; CV2 = 19.24%; CV3 = 17.89%; CV4 = 12.48%). The plot of CV1 versus CV2 shows that fossorial and aquatic forms can be readily distinguished, while there is some degree of overlap among the other groups (figure 4). The plot of CV1 versus CV3 separates fossorial and semi-aquatic forms, with the notable exception of *Pantherophis guttatus*, which, despite being classified as a generalist, still falls inside the 90% confidence ellipsoid of semi-aquatic forms. In this plot arboreal forms are also largely separated from the other groups. The plot of CV2 versus CV4 is the best at separating generalist and aquatic taxa.

As expected, the most apparent morphological changes are described by the first two canonical variates, which show that taxa with high positive values of CV1 tend to have a more well developed anterior semicircular canal and lateral ampulla (the size of which is also correlated with that of the anterior ampulla). These taxa also have a relatively smaller sacculus, and consequently a lateral semicircular canal that does not extend as far laterally as in taxa with negative CV1 scores (e.g. fossorial forms). CV2 is the axis that best separates aquatic forms from the rest. The shape change associated with positive values can be described as a general mediolateral compression of the whole inner ear. CV3 is the axis that best separates semi-aquatic forms from the rest, and changes in the positive direction along this axis can be summarized as a general increase in the size of the sacculus and lateral bulging of the horizontal semicircular canal. This is similar to the change associated with negative values of CV1, but unlike the latter, the anterior semicircular canal and the lateral ampulla are not reduced in size as much. Generalist forms tend to have negative values of CV4, and values of CV2 of less than 2, which means that their inner ears are not mediolaterally compressed (unlike aquatic forms, which show values of CV2 of more than 4), and at the same time they tend to have an anterior semicircular canal that presents a more pronounced concavity in lateral view (figure 4).

The CVA jacknife cross-validation results, obtained with the R package Morpho v. 2.4.1.1 [12], are indicative of a very strong ecological signal in the data, since the overall classification accuracy was 98.75%, with only one generalist species misclassified as semi-aquatic (Kappa statistic = 0.9837).

## 4. Discussion

In a novel recent contribution to the debate on the ecological context for the origin of snakes, Yi & Norell [2] provided an argument in favour of the burrowing origin of this group. The main points they raised and their line of reasoning can be summarized as follows: (i) *Dinilysia* is a stem snake close to the most recent common ancestor of living snakes according to most phylogenetic analyses (e.g. [18–20]); (ii) *Dinilysia* exhibits an inner ear morphology that is very similar to that of certain burrowing squamates (notably the enlarged spherical sacculus; termed ‘vestibule’ in [2]); (iii) according to their predictive model, both *Dinilysia* and the reconstructed ‘hypothetical ancestor of crown snakes’ are classified as burrowing forms based on a comparison with modern species of known ecology; (iv) burrowing is a predominant lifestyle in the most basal lineages of crown snakes; (v) therefore, snakes must have had a burrowing origin.

Yi & Norell's work [2] is a valuable and important contribution to the debate on snake origins; however, their dataset included only a relatively small sample of squamate diversity (43 extant species), most of which were burrowers (20 out of 43); they only considered three broad ecological categories (aquatic, burrowing, generalist); and their landmarking scheme focused only on shape changes involving the distance between the sacculus and the horizontal semicircular canal (they placed no landmarks or semilandmarks on anterior and posterior semicircular canals).

In contrast, the dataset analysed here includes almost twice as many taxa, subdivided the species in five traditional ecological categories (noting as well the difficulties with the fossorial category) to improve the precision and discriminatory power of our analysis, and adopted a new landmarking scheme that takes into account not only the size of the sacculus but also the shape of all three semicircular canals and the ventral extent of the lagena. We did not estimate the shape of the ‘hypothetical ancestor’ of crown snakes since that is highly dependent on the taxa sampled and the phylogeny used (the exact position of *Dinilysia* is still debated (e.g. [17–20]); and the closest outgroup to snakes might be iguanians, anguimorphs or mosasauroids (e.g. [16,17,20]).

Our results place *Dinilysia* very close to the extant snake *Xenopeltis* (corroborating the results of [2]); however, we also find that certain semi-aquatic snakes, namely homalopsids, are very close to *Dinilysia* as well. This contradicts the claim by [2] that a large spherical sacculus (their ‘vestibule’) is diagnostic of burrowing forms. Such morphology can also be found in semi-aquatic snakes (not sampled in the earlier study), and is absent in some typical obligate burrowers like scolecophidians, or other semi-fossorial snakes such as *Calabaria* or *Brachyurophis*. It is also important to note that scolecophidians are widely considered to be the most basal lineage(s) of crown snakes both historically [38] and in recent phylogenetic analyses [16–20]. If this is indeed an accurate phylogenetic position for these snakes, then contra [2], it might be expected that *Dinilysia* would display an inner ear morphology similar to scolecophidian snakes if burrowing was indeed primitive for snakes. The sharp differences between the inner ears of *Dinilysia* and scolecophidians are not easily explicable if all are considered to share a common burrowing ancestry.

Our Procrustes ANOVA (both phylogenetic and non-phylogenetic) and CVA found a strong correlation between shape and habitat groups, and our classification test placed *Dinilysia* among fossorial s.l. forms (note: this ecological group includes both semi-fossorial and obligate burrowers) with the highest (but overall very low; 4.4–5.8%) probability (in agreement with [2]). Our new study raises two important caveats. (i) *Dinilysia* occupies a portion of morphospace outside the clouds of all five ecological categories of living squamates. While the classification test favours the fossorial category over all other categories (0.044–0.058), the semi-aquatic category is not far behind (0.018–0.021), and the *most* likely placement is outside of all five categories (0.97–0.98). (ii) It should be kept in mind that the data are affected by phylogenetic signal (contra [2]), which might cause the grouping of *Dinilysia* with *Cylindrophis* and *Xenopeltis*, which are all relatively basal snakes under some phylogenetic hypotheses (i.e. similarity due to common ancestry, and not similarity due to convergence and convergent ecologies), although this possibility seems to be excluded by the results of our phylogenetic PCA (figure 3), provided that our adoption of the Brownian motion model of evolution is a reasonable enough approximation. The resemblance between the inner ear of *Dinilysia* and that of the semi-aquatic homalopsids, however, cannot be due to close phylogenetic relationship, because homalopsids are very derived snakes nested within the clade Colubroidea [16]. Such resemblance is more likely due to convergence, and if so, the habitat preference implied is semi-aquatic to semi-fossorial, not pure burrowing.

Moreover, *Xenopeltis* and *Cylindrophis*, the two snakes that we retrieved closest to *Dinilysia* in the plots of PC1 versus PC2 and PPC1 versus PPC2 (figures 2 and 3), are traditionally classified as fossorial, but both species also have an affinity for very wet habitats; *Cylindrophis* is known to feed on eels, and *Xenopeltis* is commonly found in swamps and rice fields [35]. The semi-aquatic, cryptozoic homalopsid snake *Myron* is also very close to *Dinilysia* (both in PC1 versus PC2 and PPC1 versus PPC2)*.* All these species have an ecological preference for wet environments as well as burrows (crab burrows in the case of *Myron* [36]), raising the likelihood that *Dinilysia* had similar habits. Interestingly, *Loxocemus*, which is also placed close to *Dinilysia* both in PC1 versus PC2 and in PPC1 versus PPC2, is another of those taxa generically classified as ‘fossorial’, but which in truth is not an active burrower and simply hides in loose soil and leaf litter [35].

In the same plots (PC1 versus PC2 and PPC1 versus PPC2), terrestrial active burrowers (e.g. the uropeltid *Teretrurus*, scolecophidians, *Eryx, Calabaria*) are all farther away from *Dinilysia* than are *Xenopeltis*, *Loxocemus*, *Cylindrophis* and *Myron*.

**Figure 5.**
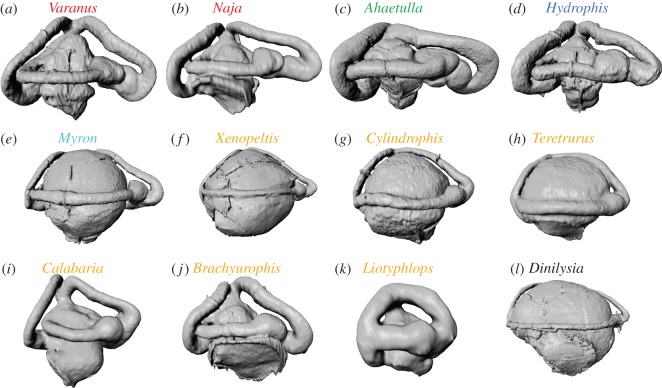
Digital endocasts of the right inner ears of various squamates in lateral view (anterior to the right). (*a*) *Varanus gilleni* (SAMA R32164), a generalist lizard; (*b*) *Naja siamensis* (SAMA R63784), a generalist snake; (*c*) *Ahaetulla prasina* (SAMA R22443), an arboreal snake; (*d*) *Hydrophis platurus* (FMNH 16927), a fully aquatic snake; (*e*) *Myron richardsonii* (SAMA R24824), a semi-aquatic snake; (*f*) *Xenopeltis unicolor* (SAMA R36861), a semi-fossorial snake with a preference for wet, swampy habitats; (*g*) *Cylindrophis ruffus* (SAMA R12956), a fossorial snake with a preference for tropical rainforest close to water bodies, where it hunts for eels; (*h*) the uropeltid snake *Teretrurus sanguineus* (NMV 8385), a truly fossorial snake (i.e. digs burrows); (*i*) *Calabaria reinhardtii* (FMNH 117833), a fossorial snake in loose soil and leaf litter; (*j*) *Brachyurophis australis* (SAMA R131571), a fossorial snake in loose soil and sand; (*k*) the scolecophidian snake *Liotyphlops beui* (SAMA R40142), a truly fossorial snake (i.e. digs burrows); (*l*) *Dinilysia patagonica* (MACN RN-1014), an extinct snake with unknown ecological preference. Note how diverse is the morphology of the inner ear of fossorial (s.l.) taxa. Names follow the same colour coding used in the other figures. For institutional abbreviations see electronic supplementary material S1, table S1. Images are not to scale.

Figure 5 summarizes the main observations about the shape of the inner ear in the examined taxa. Generalist forms tend to have a sacculus that is similar in size to the lagena (figure 5*a*,*b*), and anterior and posterior semicircular canals that are also comparable in size; arboreal forms tend to be characterized by a relatively larger and stouter anterior semicircular canal (figure 5*c*); while aquatic taxa tend to resemble generalists (figure 5*d*), and do not appear to have diverged considerably in the geometry of their inner ear, something that is highlighted by the extensive overlap between these two categories in the principal component analyses (figures 2 and 3). Semi-aquatic snakes, on the other hand, especially those belonging to the Homalopsidae, tend to have a relatively much larger sacculus (figure 5*e*), a condition very similar to that observed in some fossorial and semi-fossorial forms (figure 5*f–h*). It is important to note, however, that not all fossorial and semi-fossorial snakes share an expanded sacculus. For example this condition is not observed in *Calabaria reinhardtii* (figure 5*i*), *Brachyurophis australis* (figure 5*j*) or scolecophidian snakes, e.g. *Liotyphlops beui* (figure 5*k*). Among the illustrated species, the inner ear of *Dinilysia patagonica* (figure 5*l*) certainly resembles most closely those of *Myron* (figure 5*e*), *Xenopeltis* (figure 5*f*), and *Cylindrophis* (figure 5*h*), a resemblance that is supported by the results of our principal component analyses. Such resemblance cannot be attributed to an exclusively burrowing ecology, since *Myron* is semi-aquatic, and not an active terrestrial burrower. Moreover, the link between a large sacculus and burrowing habits is far from unequivocal, as illustrated by snakes like *Calabaria*, *Liotyphlops* and *Brachyurophis*. It is also important to point out that, contrary to [2], a large fenestra ovalis is not necessarily indicative of fossorial habits either, since this feature is present in the generalist snake *Naja siamensis* (figure 5*b*), and is absent in scolecophidian snakes, which are all obligate, active burrowers.

Yi & Norell [2] stated that all most basal lineages of crown snakes are fossorial and this should be taken as indication that snakes likely had a burrowing origin. This argument has a long and venerable history, dating back at least to [38]. However, we should keep in mind that snakes originated in the Mesozoic, where they were already quite diverse and occupied various niches inclusive of both terrestrial and aquatic environments [39,40]. The fact that living lineages of presumed basal snakes are all fossorial or semi-fossorial may be the result of ecological screening through the last mass extinction at the end of the Cretaceous (K-Pg boundary), where burrowing lineages of snakes may have survived thanks to their habitat preference [41]. Any inference on the ancestral ecology of snakes that is based only on the lineages that made it past the last mass extinction would thus be unavoidably biased.

There is strong evidence from bone histology and microanatomy that several stem ophidians from the Upper Cretaceous (e.g. *Eupodophis*, *Haasiophis*, *Mesophis*, *Pachyophis*, *Pachyrhachis*, *Simoliophis*) were aquatic [42–44], and this may be indicative of an aquatic ancestry of the group, as suggested by several previous studies (e.g. [45–48]). The semi-aquatic habits suggested for *Dinilysia* in this study may well be a reflection of the morphological adaptations from an aquatic ancestry, possibly overprinted by a new terrestrial ecology. A mixture of phylogenetic constraints (aquatic morphology) and partial adaptation to a terrestrial ecology (semi-burrowing morphology) may also be responsible for the placement of the inner ear of *Dinilysia* in a portion of morphospace that is quite unique and different from that of any of the extant ecological groups. Situations where fossil species fall outside all groups defined by modern taxa and their ecologies are quite common (e.g. [49,50]), and simply mean that inferences about the palaeoecology of these fossils need to be done with alternative possibilities carefully noted and integrated with other sources of data.

## 5. Conclusion

Our study shows that the large spherical sacculus, a hallmark of burrowing habits according to [2], is not unique to fossorial squamates, but is also observed in certain semi-aquatic forms. Moreover, such a shape is not typical of all burrowers, since many typical burrowers/semi-burrowers (e.g. scolecophidians, *Calabaria*, *Brachyurophis*) lack an enlarged sacculus. We find that, with the exception of *Loxocemus*, all the living snakes with inner ears most similar to *Dinilysia* have an ecological preference that includes both burrows and wet habitats (although *Myron* is not an active burrower). Given this, semi-aquatic habits cannot be rejected for the fossil snake *Dinilysia*, and this may or may not be the result of a more fully aquatic heritage. *Dinilysia* may well have had a predilection for burrows (not necessarily actively excavated) as well as aquatic environments, most likely fluvial, as suggested by the lithotypes associated with the specimens, i.e. sandstones and conglomerates. Interestingly, burrows are also common in the same unit [29,51,52]. Such ecology would not be too dissimilar from that of living homalopsids, which have been suggested to be a good analogue for the ancestral snake [53].

Trying to make predictions of the ecological driver that spurred the origin of a very diverse group of organisms with a poor fossil record can only be an extremely difficult task. Such questions cannot be conclusively answered by looking at a single fossil species (in this case *Dinilysia*), which may or may not have been representative of the ancestral ecology of the group. This is especially evident if we consider that the oldest stem snakes appeared in the Late Jurassic, about 80 million years before *Dinilysia*, with an already broad ecological and geographical distribution [40], and that it took much less than that (i.e. 25 million years [54]) for Australasian elapid snakes to radiate into all five ecological categories (generalist, fossorial, semi-aquatic, aquatic, arboreal). It is of course possible that snakes evolved from among lizards as a result of an adaptation to a purely burrowing, or an exclusively aquatic environment, but either of these scenarios may be too simplistic.

Moreover, the ecological factor(s) that drove the evolution of the unique skull anatomy of snakes (notably cranial kinesis) may be different from what drove body elongation and limb loss [40]. In fact, cranial specializations and axial elongation are not necessarily coupled, since many limbless and elongated lizards lack evidence of cranial specialization (e.g. cranial anatomy of limbless anguids, such as *Ophisaurus* and *Pseudopus*, is mostly conserved regardless of limb loss and body elongation [55,56]), while the fossil stem amphisbaenian *Cryptolacerta hassiaca* shows cranial specializations for burrowing but no body elongation nor limb loss [57].

As is evident in modern snakes, limbless locomotion can be very effective in multiple environments: above ground, through soil and litter, in the trees, in the littoral zone, or in deep water. Regardless of the particular environment in which the first snakes evolved, we might expect that once limblessness had been selected for one environment, very rapid radiation into other environments could well have followed, making it difficult if not impossible to infer the original selective context. To further complicate matters, if limb loss (or at least, the final stages of limb loss) has occurred many times throughout snake evolution, as suggested by the fossil record and comparative morphology of extant forms [58], then the original selective context linking limb reduction to any one environment might be virtually impossible to discover. Indeed, different lineages—or even the same lineage at different points in time—might have been exposed to different selective contexts.

## Supplementary Material

Supplementary material S1 - Table S1

## Supplementary Material

Supplementary material S2

## Supplementary Material

Supplementary material S3 - Figures S1-S4

## Supplementary Material

Supplementary material S4 - Table S2

## Supplementary Material

Supplementary material S5

## Supplementary Material

Supplementary material S6 - Table S3

## Supplementary Material

Supplementary material S7

## Supplementary Material

Supplementary material S8 - Table S4

## Supplementary Material

Supplementary material S9

## Data accessibility

Additional resources are available as supplementary data at: http://dx.doi.org/10.5061/dryad.ft0qm [59].
