# Supplementary material for: The morphology of the inner ear of squamate reptiles and its bearing on the origin of snakes
Source: R Soc Open Sci. 2017 Aug 23;4(8):170685. doi: 10.1098/rsos.170685 (PMC5579127; doi:10.1098/rsos.170685)
Supplement: Supplementary material S3 - Figures S1-S4 [file rsos170685supp3.pdf]

Royal Society Open Science

The morphology of the inner ear of squamate reptiles and its bearing on the origin of snakes

Alessandro Palci[1,2]*, Mark N. Hutchinson[1,2,3], Michael W. Caldwell[4], and Michael S. Y. Lee[1,2]

1 South Australian Museum, Adelaide, SA, Australia.

2 College of Science and Engineering, Flinders University, Adelaide, SA, Australia.

3 School of Biological Sciences, University of Adelaide, Adelaide, SA, Australia.

4 Department of Biological Sciences, University of Alberta, Edmonton, AB, Canada.

*Email: alessandro.palci@flinders.edu.au

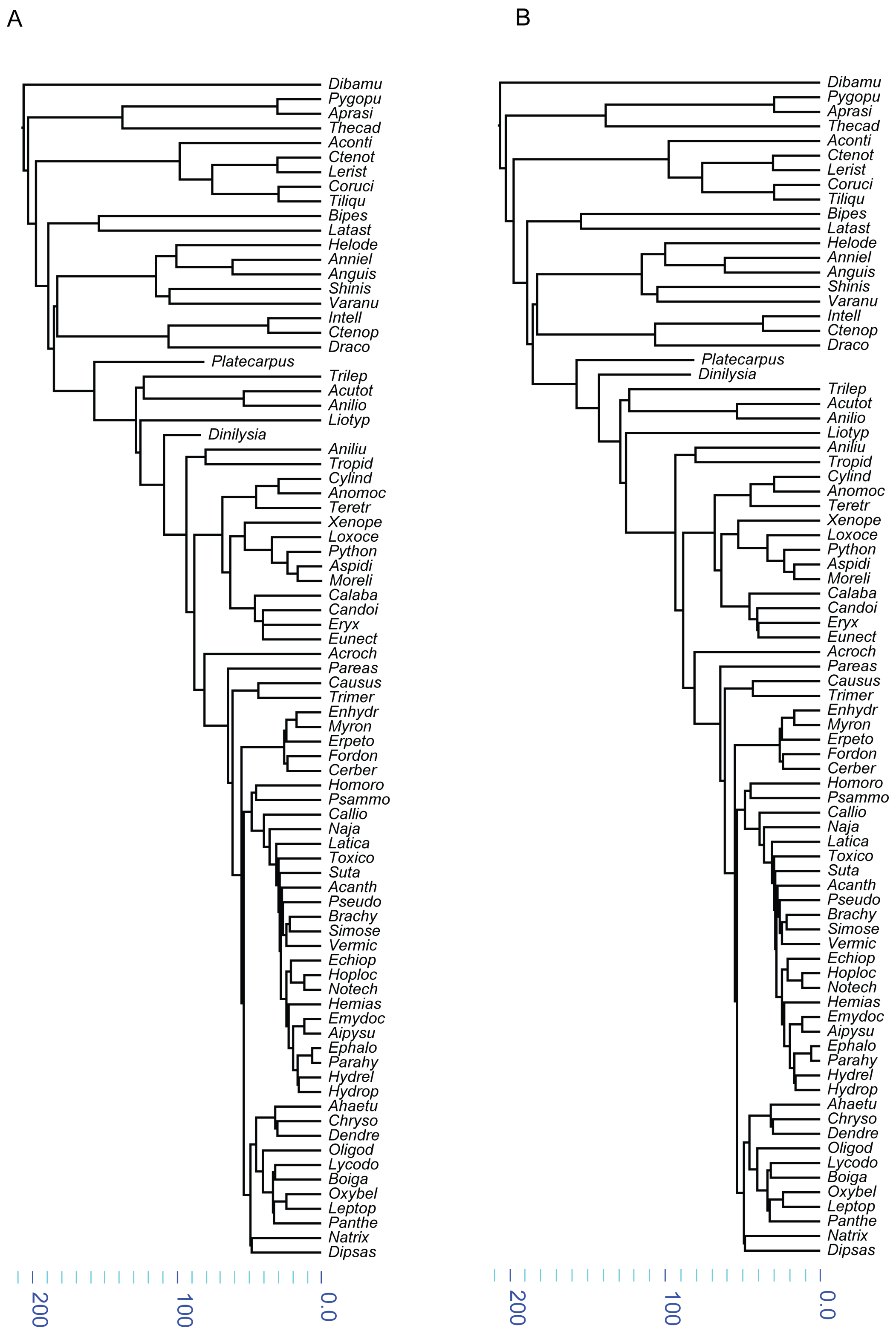

Figure S1. Phylogenetic trees used for phylogenetic analyses that were inclusive of fossil taxa. A, tree topology with *Dinilysia patagonica* as a stem alethinophidian; B, tree topology with *Dinilysia patagonica* as a stem ophidian. Branch lengths are proportional to time (millions of years). See main text for further details. For correspondence between abbreviations and taxonomic names see supplementary material S4, Table S2.

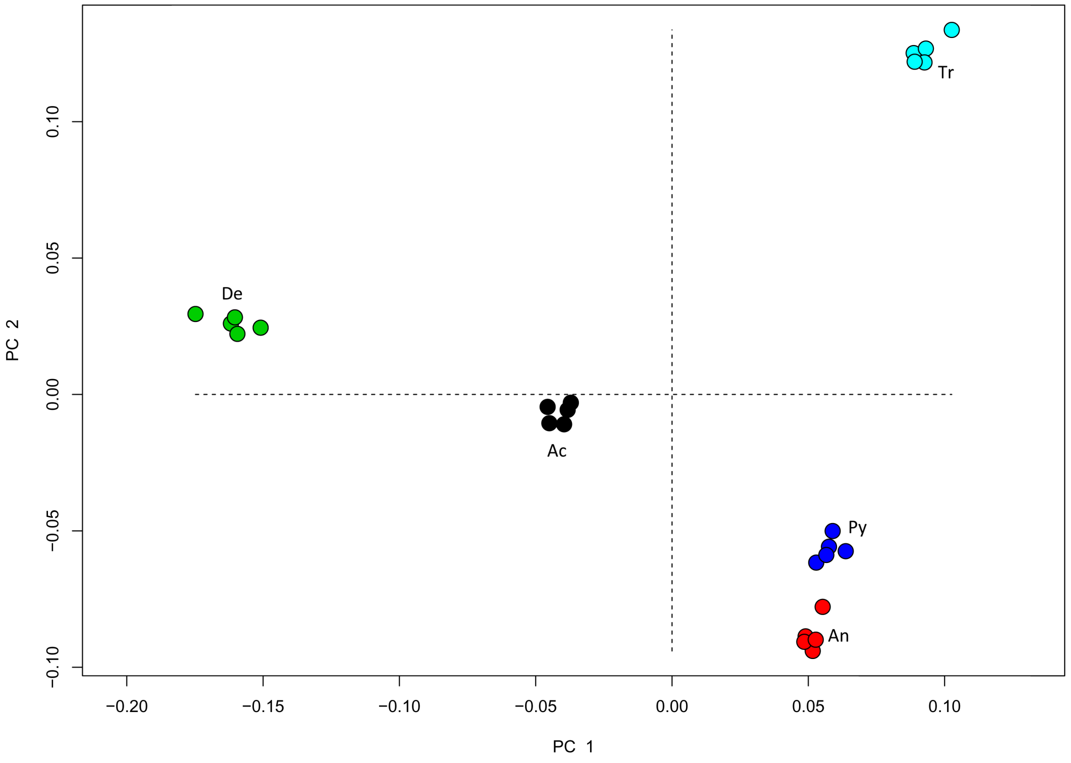

Figure S2. Testing accuracy of landmarking procedure. Principal components analysis of five randomly selected taxa that were landmarked five times each. Abbreviations: Ac, *Acanthophis antarticus*; An, *Anilius scytale*; De, *Dendrelaphis calligastra*; Py, *Python molurus*; Tr, *Trilepida dimidiatum*.

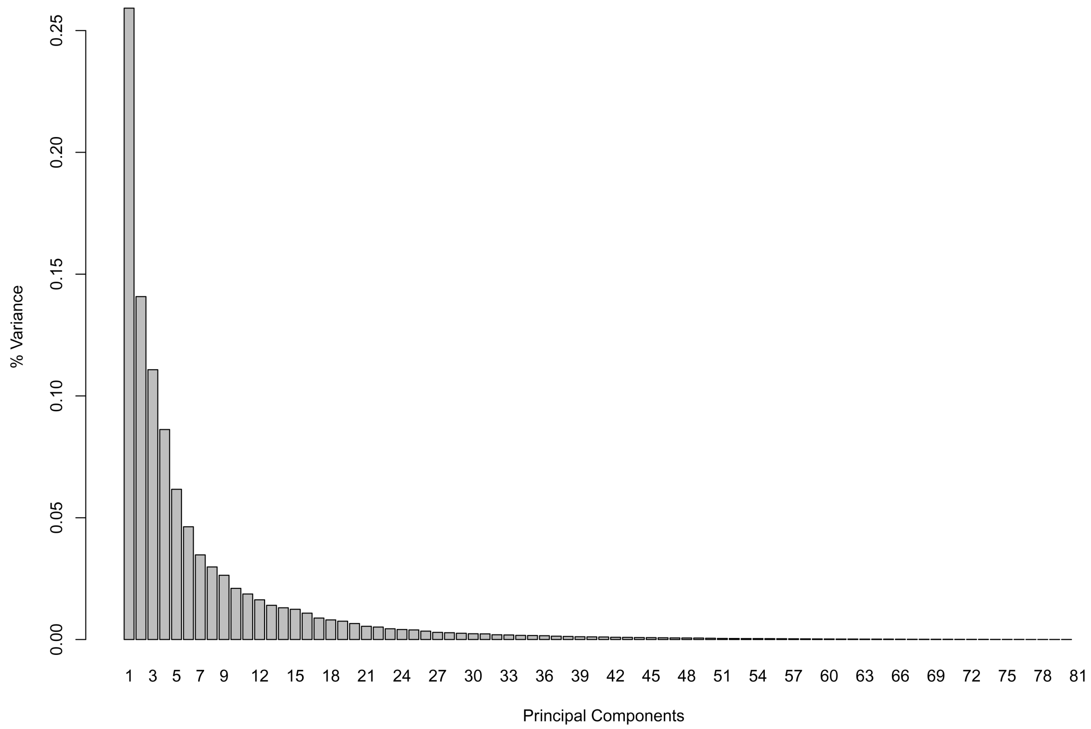

Figure S3. Distribution of variance explained by each principal component in the ordinary PCA of 81 taxa.

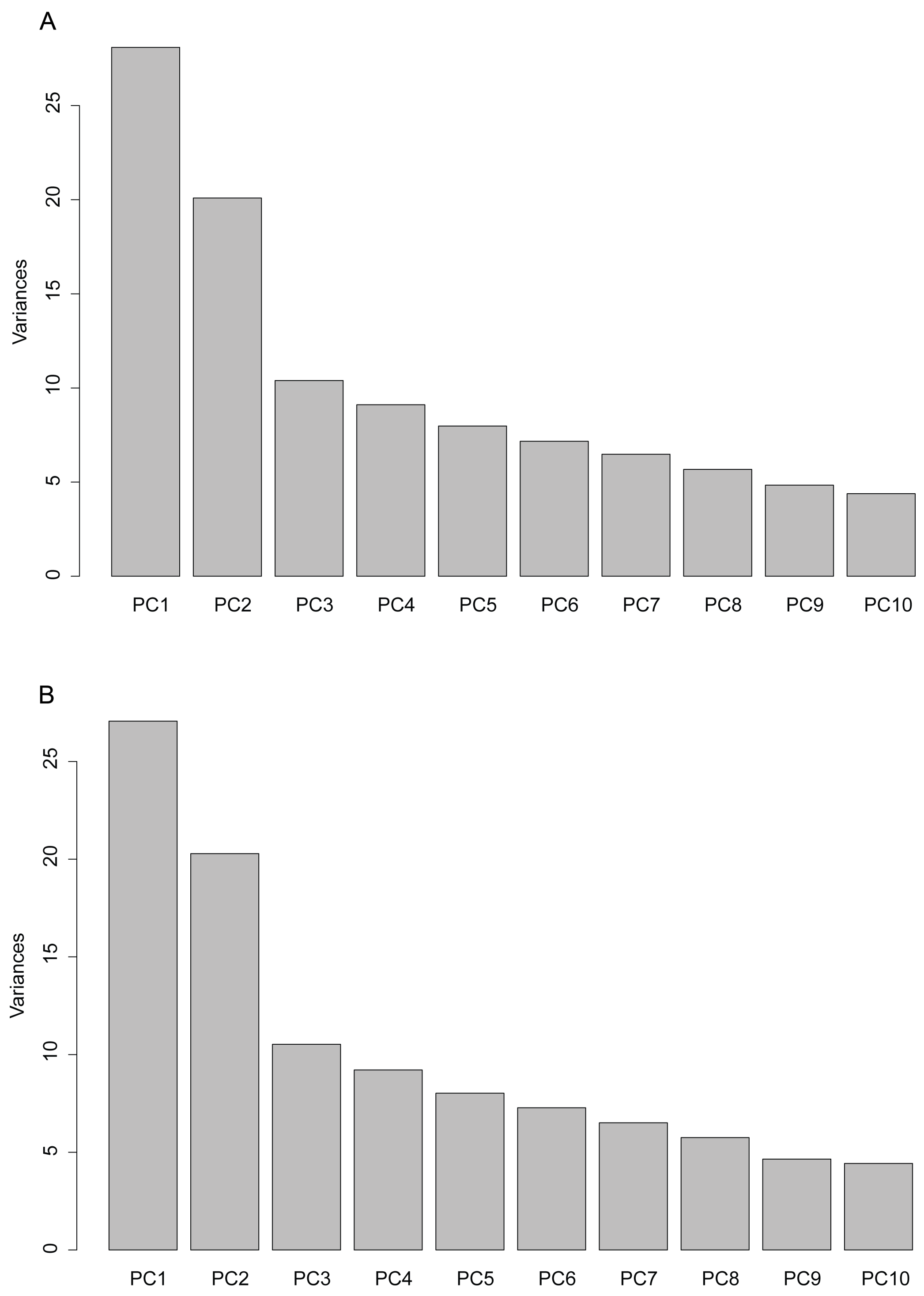

Figure S4. Distribution of variance explained by the first 10 principal components in the phylogenetic PCAs of 81 taxa. A, screeplot for the phylogenetic PCA based on the tree that had *Dinilysia* as a stem alethinophidian; B, screeplot for the phylogenetic PCA that had *Dinilysia* as a stem ophidian.
