# Supplementary material for: The morphology of the inner ear of squamate reptiles and its bearing on the origin of snakes
Source: R Soc Open Sci. 2017 Aug 23;4(8):170685. doi: 10.1098/rsos.170685 (PMC5579127; doi:10.1098/rsos.170685)
Supplement: Supplementary material S7 [file rsos170685supp7.html]

 
RGL model


You must enable Javascript to view this page properly.

  
Drag mouse to rotate model. Use mouse wheel or middle button
to zoom it.

---

  
Object written from rgl 0.96.0 by writeWebGL.
